# Sodium butyrate protects against lipopolysaccharide-induced liver injury partially via the GPR43/ β-arrestin-2/NF-κB network

**DOI:** 10.1093/gastro/goaa085

**Published:** 2020-11-22

**Authors:** Qian-Jiang Luo, Mei-Xing Sun, Yun-Wei Guo, Si-Wei Tan, Xiao-Ying Wu, Kodjo-Kunale Abassa, Li Lin, Hui-Ling Liu, Jie Jiang, Xiu-Qing Wei

**Affiliations:** 1 Department of Gastroenterology, The Third Affiliated Hospital of Sun Yat-sen University, Guangzhou, Guangdong, P. R. China; 2 Department of Gastroenterology, The Eighth Affiliated Hospital of Sun Yat-sen University (Shenzhen Futian Hospital), Shenzhen, Guangdong, P. R. China

**Keywords:** sodium butyrate, short-chain fatty acids, lipopolysaccharide-induced liver injury, G-protein-coupled receptor 43, β-arrestin-2, NF-κB

## Abstract

**Background:**

Butyrate acts as a regulator in multiple inflammatory organ injuries. However, the role of butyrate in acute liver injury has not yet been fully explored. In the present study, we aimed to investigate the association between butyrate and lipopolysaccharide (LPS)-induced acute liver injury and the signaling pathways involved.

**Methods:**

LPS-induced acute liver injury was induced by intraperitoneal injection of LPS (5 mg/kg) in G-protein-coupled receptor 43 (GPR43)-knockout (KO) and wild-type female C57BL/6 mice. Sodium butyrate (500mg/kg) was administered intraperitoneally 30 min prior to LPS exposure. Liver injury was detected by serum markers, tissue morphology, and terminal deoxynucleotidyl transferase-mediated dUTP nick end labeling (TUNEL). Pro-inflammatory-factor levels were detected by enzyme-linked immunosorbent assay and real-time polymerase chain reaction (RT-PCR). Cell models were first treated with sodium butyrate (4 μmol/mL), followed by LPS (1 μg/mL) half an hour later in GPR43 small interfering RNA (siRNA)-transfected or control RAW264.7 cells. Cell-inflammation status was evaluated through detecting pro-inflammatory-factor expression by RT-PCR and also through checking toll-like receptor 4/nuclear factor-κB (TLR4/NF-κB)-element levels including TLR4, TRAF6, IKKβ, IкBα, phospho-IкBα, p65, and phospho-p65 by Western blot. The interaction between GPR43 and β-arrestin-2 was tested by co-immunoprecipitation.

**Results:**

Sodium butyrate reversed the LPS-induced tissue-morphology changes and high levels of serum alanine aminotransferase, aspartate transaminase, myeloperoxidase, TUNEL, and pro-inflammatory cytokines such as tumor necrosis factor-α and interleukin-6. The ameliorating effect of sodium butyrate was weakened in GPR43-KO mice and GPR43 siRNA RAW264.7 cells, compared with those of GPR43-positive controls. Sodium butyrate downregulated some elements of the TLR4/NF-κB pathway, including phospho-IκBα and phospho-p65, in RAW264.7 cells. Increased interactions between GPR43 and β-arrestin-2, and between β-arrestin-2 and IкBα were observed.

**Conclusion:**

Sodium butyrate significantly attenuated LPS-induced liver injury by reducing the inflammatory response partially via the GPR43/β-arrestin-2/NF-κB signaling pathway.

## Introduction

Diet goes beyond nutritional value and profoundly influences human health. Lifestyle changes in recent years have paralleled the increasing incidence of various gastrointestinal and metabolic diseases, such as inflammatory bowel diseases (IBDs), intestinal cancers, and metabolic syndrome. Short-chain fatty acids (SCFAs) are produced from dietary fibers through fermentation by the microbiota in the lower intestine and are indigestible by intestinal enzymes [1]. Locally, the majority of SCFAs are used as fuel for colonocytes, while the rest exit the colonic tissue into portal circulation and act as primary substrates for hepatic metabolism [2]. Among SCFAs, four-carbon butyrate is the least abundant but has the strongest physiological function and has been extensively investigated as a key player that suppresses pathological metabolism, inflammation, and carcinogenesis throughout the entire body [3, 4]. 

Sepsis is a critical disease with a mortality rate of ≤50% [5]. Its pathogenesis primarily relates to macrophages and endothelial cells producing a large number of cytokines, such as tumor necrosis factor (TNF)-α, interleukin (IL)-1, IL-6, and IL-8 [6]. Endotoxemia is the most common cause of sepsis. It is a pathophysiological condition in which large amounts of endotoxin release into the blood due to bacterial lysis or endotoxin-contaminated-fluids injection. Liver injury induced by endotoxin is the pathophysiological basis of various liver diseases [7, 8]. The essence of endotoxin is lipopolysaccharide (LPS), which is the major structural component of the outer membrane of Gram-negative bacteria. LPS-induced liver inflammation [9] is a result of interactions of multiple cytokines, chemokines, and cell-death molecules. Inflammation is initiated by liver-resident macrophage Kupffer cells [10] and involves TNF-α, Fas/Fas ligand, and perforin/granzyme cell-death pathways. The crosstalk of antigens within immune cells and modes of cell death leads to the exacerbation of liver injury [11]. To date, LPS-induced liver injury has been associated with the pathology of various chronic liver diseases [8]. For instance, evidence shows that, even in human immunodeficiency virus (HIV) patients with well-controlled viral-replication levels, increased circulating LPS is still a major driver of liver failure resulting in morbidity and mortality [12]. 

Nuclear factor-κB (NF-κB) is a well-known major inflammatory factor that pushes the inflammatory process forward to eventually cause potential tissue damage and is activated in LPS-induced liver injury [13]. Numerous independent studies have shown that SCFAs restrain NF-κB-signaling-pathway activity to regulate inflammatory responses [14]. SCFAs serve as a group of endogenous agonists of G-protein-coupled receptor 43 (GPR43) [2], which is a member of the G-protein-coupled receptors (GPCRs) that are famous for cell-signal transduction. GPR43 is highly expressed in intestinal epithelial cells and immune cells [15–17]. Previous studies have shown that, as the most notable SCFA target, GPR43 mediates the protective effects of SCFAs in the inflammatory process [18, 19]. β-Arrestin-2 is widely expressed in the cytoplasm of cells and serves as a scaffold protein for a wide range of GPCRs [20]. It participates in GPCR desensitization and internalization, and contributes to the downstream formation of the signaling compound following GPCR activation [21]. Of note, β-arrestin-2 also has a beneficial effect in inflammation through various paths, mainly by reducing NF-κB nuclear translocation and eventual activation [22–24]. Given this background, the influence of sodium butyrate on LPS-induced mouse liver injury and inflammation, and its involvement in the NF-κB pathway via GPR43 and β-arrestin-2 were investigated in our studies.

## Materials and methods

### Reagents and chemicals

LPS (Cat. L2630), sodium butyrate (Cat.B5887), and TRIzol reagent (Cat. T9424) were obtained from Sigma (St Louis, MO, USA). Dulbecco’s modified Eagle’s medium (DMEM; Cat. C11995500B), fetal bovine serum (Cat. 10270–106), penicillin and streptomycin (Cat. 15140122), and trypsin (Cat. 25200–056) were obtained from Gibco (Rockville, MD, USA). The real-time PCR Master Mix kit-SYBR Green (Cat. AQ141-04) was obtained from TransGen (Beijing, China). Small interfering RNA (siRNA)-GPR43 was designed by GenePharma (Shanghai, China). Anti-glyceraldehyde-3-phosphate dehydrogenase antibody (GAPDH; Cat. sc-25778), horseradish peroxidase (HRP)-labeled goat anti-rabbit immunoglobulin G (IgG) secondary antibody (Cat. sc-2004), anti-p65 (Cat. sc-372), anti-phospho-IkBα (Cat. sc-8404), anti-IkBα (Cat. sc-371), and anti-TRAF6 (Cat. sc-7221) antibodies were all obtained from Santa Cruz (Santa Cruz, CA, USA). Anti-GPR43 (Cat. ABC299) antibodies were obtained from Merck (Darmstadt, Germany). Anti-myeloperoxidase (MPO) primary antibody (Cat. ab9535), anti-β-arrestin-2 (Cat. ab54790), and anti-phospho-p65 (Cat. ab86299) were obtained from Abcam (Cambridge, MA, USA). Lipofectamine 3000 (Cat. L3000-015) was purchased from Invitrogen (Carlsbad, CA, USA). The First Strand cDNA Synthesis Kit ReverTra Ace-α-^TM^ (Cat. FSK-100) was obtained from Toyobo (New York, NY, USA). Alanine transaminase (ALT; Cat.CSB-E16539m) and aspartate transaminase (AST; Cat.CSB-E12649m) enzyme-linked immunosorbent assay (ELISA) kits were all obtained from Cusabio (Wuhan, Hubei, China). The Pierce Classic IP Kit (Cat. 26146) was obtained from Thermo Scientific (Rockford, AL, USA).

### Mouse-model preparation

Forty female C57BL/6 mice aged 6–8 weeks and weighing 18–22 g were purchased from Guangdong Medical Laboratory Animal Center. Because male mice often fight with each other, which makes them more vulnerable to liver injury [25, 26], in this study, we used only female mice. The original GPR43^+/−^ heterozygous C57BL/6 mice were purchased from Bioray Laboratories Inc. (Shanghai, China) and were bred, propagated, and identified at the Institutional Animal Care and Use Committee of Sun Yat-sen University (Guangzhou, Guangdong, China). All experiments were approved by the Institutional Animal Care and Use Committee of Sun Yat-sen University (certification no.: IACUC-F3-17–1004). Animal studies were performed in compliance with the Animal Research: Reporting of In Vivo Experiments (ARRIVE) guidelines [27, 28]. Forty female C57BL/6 mice were randomly divided into 4 groups of 10 mice. Female GPR43 knockout (KO) animals and wild-type (WT) littermates were randomly divided into four groups of six mice each. Animal models were established by intraperitoneal injection of sodium butyrate (500 mg/kg) or normal saline as a pretreatment, followed by intraperitoneal injection of LPS (5 mg/kg) or normal saline 30 min later. The control-group mice were injected intraperitoneally with normal saline during both steps. After 6 h, the mice were anesthetized by intraperitoneal injection of a mixed solution of ketamine and xylazine. Blood samples were collected from the retrobulbar plexus and the serum was stored at –20°C before testing. The liver of each mouse was carefully isolated. Some liver tissues were immediately fixed in 10% neutral buffered formalin and the rest were stored at –80°C.

### Cell-culture and model preparation

The RAW264.7 cell line was purchased from Shanghai Institutes of Biological Sciences. The cells were cultured at 37°C in a 5% CO_2_-humidified incubator in DMEM supplemented with 10% fetal bovine serum and 1% antibiotics (penicillin and streptomycin). Cells were seeded in a six-well plate at 2 × 10^5^ cells per well for further experimentation. Transfections of cells with GPR43 siRNA RNA oligo (GPR43 siRNA) or control siRNA RNA oligo (control siRNA) were performed using Lipofectamine 3000 for 24 h. The experimental-group cells were first treated with sodium butyrate (4 μmol/mL), followed by LPS (1 μg/mL) half an hour later. The control-group cells were treated with normal saline for both of the previously mentioned steps. After 6 h, the cell lysates were collected for further analysis. The primer sequences of the siRNAs are shown in Supplementary Table 1.

### Enzyme-linked immunosorbent assay

To evaluate liver injury, the levels of ALT and AST in mouse serum were detected using ELISA kits.

### Quantitative real-time PCR

Total RNA was collected using TRIzol reagent and reverse transcription was performed with the First Strand cDNA Synthesis Kit ReverTra Ace-α-^TM^. The mRNA-expression levels of TNF-α, IL-6, IL-1β, IL-8, cyclooxygenase (COX)-2, and interferon (IFN)-γ were analysed by real-time polymerase chain reaction (RT-PCR) using a CFX Connect Real-time PCR System (BioRad; Hercules, CA, USA) with SYBR Green (TransGen). Each sample was normalized to the β-actin gene to control for unwanted sources of variation. Primer sequences of the above genes are provided in Supplementary Table 2.

### Histopathological score of liver injury

Hematoxylin and eosin (H&E) staining was performed as described in our previous study [24]. Terminal deoxynucleotidyl transferase-mediated dUTP nick end labeling (TUNEL; dUTP-digoxigenin) staining was performed [29] according to the instructions of the In Situ Cell Death Detection Kit (Roche; Basel, Switzerland). Based on Hoque’s report, apoptosis and hemorrhage scores were tested in a blinded manner in five 200× magnified fields [30]. According to the rate of hepatocyte apoptosis, apoptosis was scored from 0 to 4 per field (0: ≤1%; 1: 1%–5%; 2: 5%–10%; 3: 10%–20%; and 4: ≥20%). Hemorrhage was also scored on a 0–4 scale based on the hemorrhage rate per field (0: 0; 1: 1%–5%; 2: 5%–20%; 3: 20%–50%; and 4: ≥50%).

### Immunohistochemical staining

For immunohistochemical staining, sections were dewaxed and treated with 3% hydrogen peroxide and then boiled in a Tris/ethylenediaminetetraacetic acid (EDTA) antigen-retrieval solution (pH 6.0) for 2 min to retrieve the antigens. Non-specific antibody binding was blocked by 10% normal serum for 30 min at room temperature. Then, the sections were incubated with primary antibodies against MPO (1:200) overnight at 4°C. The target protein was detected using the 3,3′-Diaminobenzidine (DAB) staining system and finally counterstained with hematoxylin.

### Co-immunoprecipitation

Co-immunoprecipitation (Co-IP) was performed using the Thermo Scientific Pierce Classic IP Kit according to the manufacturer’s protocol. Cells were seeded in six-well plates at 2 × 10^5^ cells per well. First, we treated the cells with sodium butyrate (4 μmol/mL) or normal saline for 30 min and then collected cell lysates. In another experiment, the experimental-group cells were first treated with sodium butyrate (4 μmol/mL), followed by LPS (1 μg/mL) half an hour later. The control-group cells were treated with normal saline in those two steps. After 6 h, the lysates were collected for further analysis. Then, 20 μL resin was added to a Pierce centrifuge column and the lysates were centrifuged at 1,000 *g* for 1 min and washed. The antibody was added and incubated on the rotator for 90–120 min at room temperature. The centrifuge column was then rinsed twice and 3 μL of sodium borohydride solution was added, followed by 15 min of gentle shaking. The centrifuge column was then washed six times using the indicated washing buffer according to the manufacturer’s instructions. Immunoprecipitation antibodies or normal IgG (negative control) were added and incubated on a rotator at 4°C overnight. The centrifuge column was then rinsed six times using the indicated wash buffer and the final samples were analysed by Western blotting using the indicated antibodies.

### Western-blot analysis

We separated the proteins by electrophoresis and transferred them to polyvinylidene difluoride membranes. The membranes were then blocked for 2 h at room temperature and incubated with primary antibodies against GPR43, TLR4, TRAF6, IKKβ, IκBα, phospho-IκBα, p65, phospho-p65, and GAPDH at 4°C overnight. After rinsing, the membranes were then incubated with HRP-conjugated secondary antibodies for 2 h at room temperature. The results were visualized using an enhanced chemiluminescence system. Image-Pro Plus 6.0 software (Media Cybernetics; Rockville, MD, USA) was used for densitometry analyses.

### Statistical analysis

SPSS version 21.0 (SPSS Inc.; Chicago, IL, USA) was used for statistical analyses. The data and statistical analyses complied with the recommendations on experimental design and analysis in pharmacology [31]. The data are expressed as the mean ± SD and statistical analyses were performed using one-way ANOVA followed by Bonferroni’s comparison post-hoc tests. A value of *P *<* *0.05 was considered statistically significant.

## Results

### Sodium butyrate alleviates LPS-induced liver injury and inflammation in C57BL/6 mice

**Figure 1.**
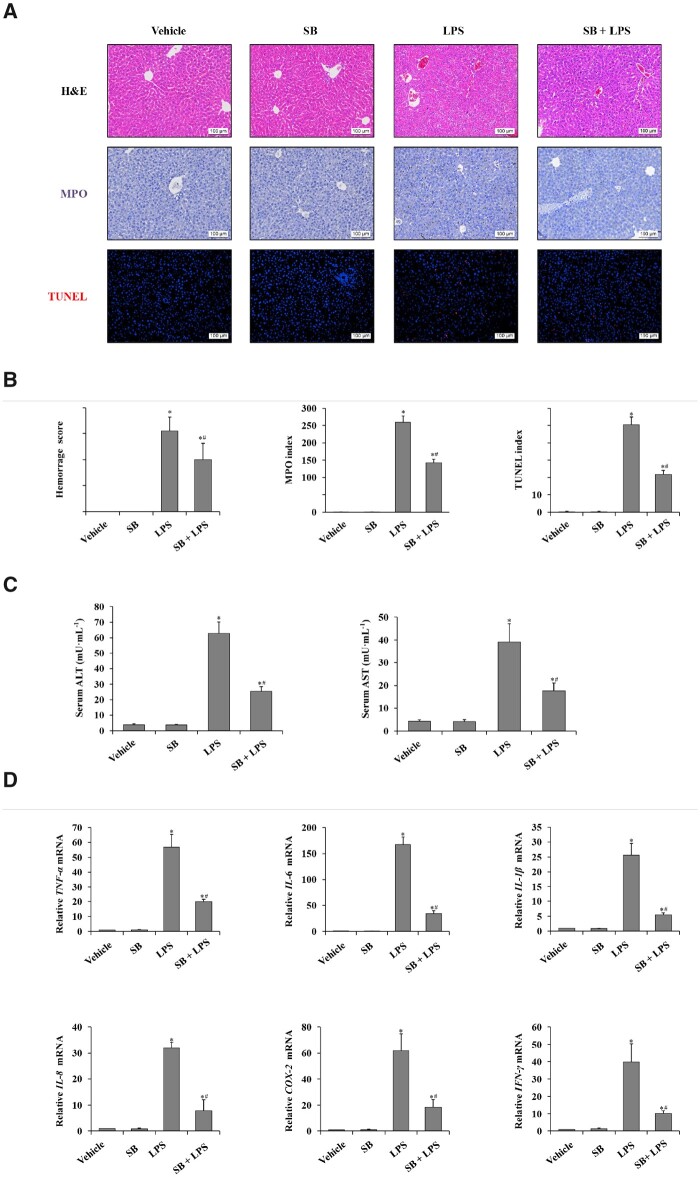
Sodium butyrate alleviates LPS-induced liver injury and inflammation. (A) H&E staining, immunohistochemical staining for MPO and TUNEL staining of liver tissue in C57BL/6 mice treated with SB, SB + LPS, LPS, and the vehicle group (magnification, ×200). (B) Histology score of hemorrhage, MPO index, and apoptosis index was calculated from TUNEL staining in C57BL/6 mice treated with SB, SB + LPS, LPS, and the vehicle group. (C) Serum levels of ALT and AST were detected using ELISA kits in female C57BL/6 mice treated with normal saline, SB, LPS, and SB + LPS. (D) RT-PCR was used to determine relative mRNA levels of TNF-α, IL-6, and IL-1β in liver tissues.^*^P <* *0.05 vs vehicle group; ^#^*P *<* *0.05 vs LPS group. Values are expressed as mean* *±* *SD (*n *=* *10 in each group). MPO, myeloperoxidase; TUNEL, terminal deoxynucleotidyl transferase-mediated dUTP nick end labeling; SB, sodium butyrate; LPS, lipopolysaccharide; ALT, alanine aminotransferase; AST, aspartate transaminase.

To investigate the role of sodium butyrate in LPS-induced liver injury, we evaluated the severity of liver injury by obtaining histological scoring, performing immunohistochemical staining for MPO and TUNEL (dUTP-biotin), and measuring the levels of serum ALT and AST. To investigate inflammation, the levels of hepatic pro-inflammatory factors were measured using RT-PCR. As shown in Figure 1, we observed that the hemorrhage score, MPO index, and apoptosis score were significantly higher in the LPS-injected group than in the vehicle control group. In the sodium butyrate + LPS group, the hemorrhage score, MPO index, apoptosis score, and serum levels of ALT and AST were significantly lower than those in the LPS group that were pretreated with normal saline (Figure 1A–C). In terms of inflammation, the mRNA levels of TNF-α, IL-6, IL-1β, IL-8, COX-2, and IFN-γ in liver tissue were significantly lower in the sodium butyrate + LPS group than those in the LPS group (Figure 1D). Based on these findings, sodium butyrate alleviated LPS-induced liver injury as well as liver inflammation in C57BL/6 mice.

### GPR43 deficiency weakened the protective effect of sodium butyrate on LPS-induced liver injury and inflammation in C57BL/6 mice

**Figure 2.**
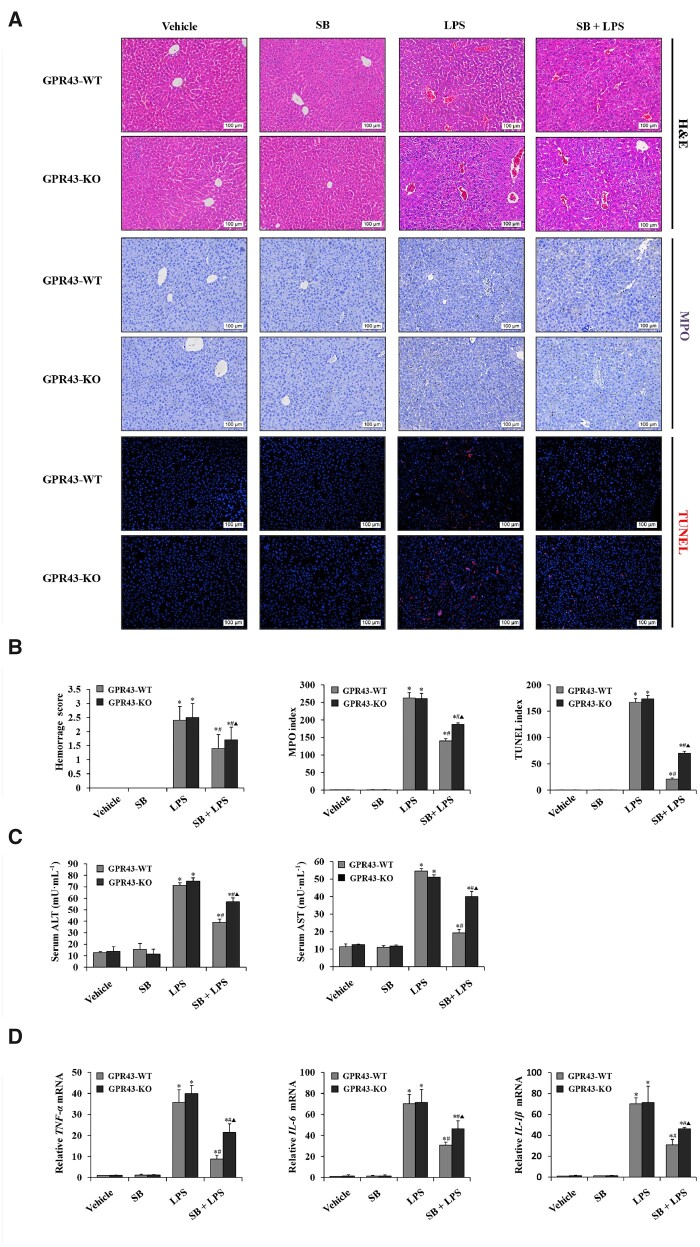
GPR43 deficiency interrupted the protection of sodium butyrate on LPS-induced liver injury and inflammation. (A) H&E staining, immunohistochemical staining for MPO, and TUNEL staining of liver tissue in female GPR43 KO mice and WT littermates treated with SB, SB + LPS, LPS, and normal saline (magnification, ×200). (B) Histology score of hemorrhage, MPO index, and the apoptosis index was calculated from TUNEL staining in female GPR43 KO mice and WT littermates treated with SB, SB + LPS, LPS, and normal saline. (C) Serum levels of ALT and AST were detected using ELISA kits. (D) RT-PCR was used to determine relative mRNA levels of TNF-α, IL-6, and IL-1β in liver tissues. ^*^P <* *0.05 vs vehicle group in the same type of mice; ^#^*P *<* *0.05 vs LPS group in the same type of mice; ▲ *P *<* *0.05 vs SB + LPS group in GPR43 WT mice. Values are expressed as mean* *±* *SD (*n *=* *6 per group). MPO, myeloperoxidase; TUNEL, terminal deoxynucleotidyl transferase-mediated dUTP nick end labeling; SB, sodium butyrate; LPS, lipopolysaccharide; ALT, alanine aminotransferase; AST, aspartate transaminase; RT-PCR, real-time polymerase chain reaction.

As butyrate can be served as endogenous agonists of GPR43, we next examined the effect of sodium butyrate in LPS-induced liver injury in GPR43-deficient mice. As shown in Figure 2, the hemorrhage score, MPO index, and apoptosis score were significantly higher in LPS-treated mice than those in the control group. A lower index was shown in the sodium butyrate + LPS group compared with that in the LPS group in the same genotype of mice. In comparison, the two different mouse genotypes in the sodium butyrate + LPS group, the hemorrhage score, MPO index, and apoptosis score were higher in GPR43 KO mice than in GPR43 WT mice (Figure 2A and B). Similar results were found regarding the levels of serum ALT and AST (Figure 2C). We evaluated the inflammation level and observed that the expression of inflammatory factors in the liver was also significantly increased due to LPS. After pretreatment with sodium butyrate, the mRNA levels of TNF-α, IL-6, and IL-1β in liver tissue were significantly decreased. In the sodium butyrate + LPS group, the levels of pro-inflammatory factors were higher in GPR43 KO mice than in GPR43 WT mice (Figure 2D). These results indicated that deletion of GPR43 weakened the effect of sodium butyrate on LPS-induced liver injury and inflammation.

### Sodium butyrate/GPR43 ameliorated liver injury and inflammation by inhibiting macrophage activation in C57BL/6 mice

**Figure 3.**
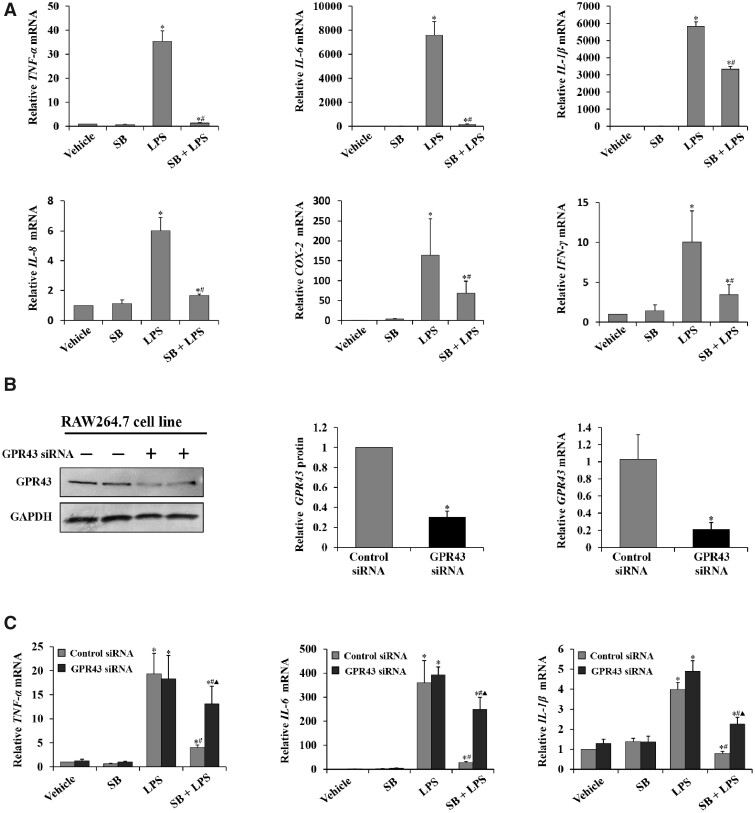
Sodium butyrate/GPR43 ameliorated liver injury and inflammation via repressing macrophage activation. (A) RT-PCR was used to determine relative mRNA levels of TNF-α, IL-6, IL-1β, IL-8, COX-2, and IFN-γ produced by RAW264.7 cell line. ^*^P <* *0.05 vs vehicle group; ^#^*P *<* *0.05 vs LPS group. Values are expressed as mean* *±* *SD (*n *=* *6 per group). (B) Expression of GPR43 in RAW264.7 cells was detected by Western blot. Levels of GAPDH are shown as a loading control; relative quantitative evaluation of the Western-blot analysis for the normalized GPR43/GAPDH was analysed from the Western-blot assay (values are expressed as mean* *±* *SD, *n *=* *6 per group, Bonferroni’s comparison post-hoc test). The relative mRNA level of GPR43 expression was detected to evaluate the siRNA interference ratio. (C) RT-PCR was used to determine relative mRNA levels of TNF-α, IL-6, and IL-1β produced by RAW264.7 cell line transfected with GPR43 siRNA RNA oligo (GPR43 siRNA) or control siRNA RNA oligo (Control siRNA). ^*^P <* *0.05 vs vehicle group in the same type of cell; ^#^*P *<* *0.05 vs LPS group in the same type of cell; *P *<* *0.05 vs SB + LPS group in control siRNA group. Values are expressed as mean* *±* *SD (*n *=* *6 per group). RT-PCR, real-time polymerase chain reaction; SB, sodium butyrate.

To confirm whether butyrate decreased the production of pro-inflammatory factors in macrophages, RAW264.7 cells, which are mouse leukemia macrophages, were utilized. As shown in Figure 3, the expression of inflammatory factors in RAW264.7 cells was significantly increased due to LPS. Similarly, in the sodium butyrate + LPS group, the mRNA levels of TNF-α, IL-6, IL-1β, IL-8, COX-2, and IFN-γ in RAW264.7 cells were significantly decreased compared with those in the LPS group (Figure 3A). To verify whether the GPR43 level was suppressed, 24 h after transfection with GPR43 siRNA, protein and mRNA expressions of GPR43 were checked and showed significant downregulation (Figure 3B). Similar to the results in the animal models, after sodium butyrate pretreatment, the mRNA levels of TNF-α, IL-6, and IL-1β in the cell line were significantly decreased. In the sodium butyrate + LPS group, the levels of pro-inflammatory factors were higher in GPR43 siRNA cells than in control siRNA cells (Figure 3C). These results indicated that deletion of GPR43 attenuated the effect of sodium butyrate on LPS-induced inflammation in macrophages.

### Sodium butyrate/GPR43 signaling inhibited macrophage activity by regulating TLR4/NF-κB p65 networks in the RAW264.7 cell line

**Figure 4.**
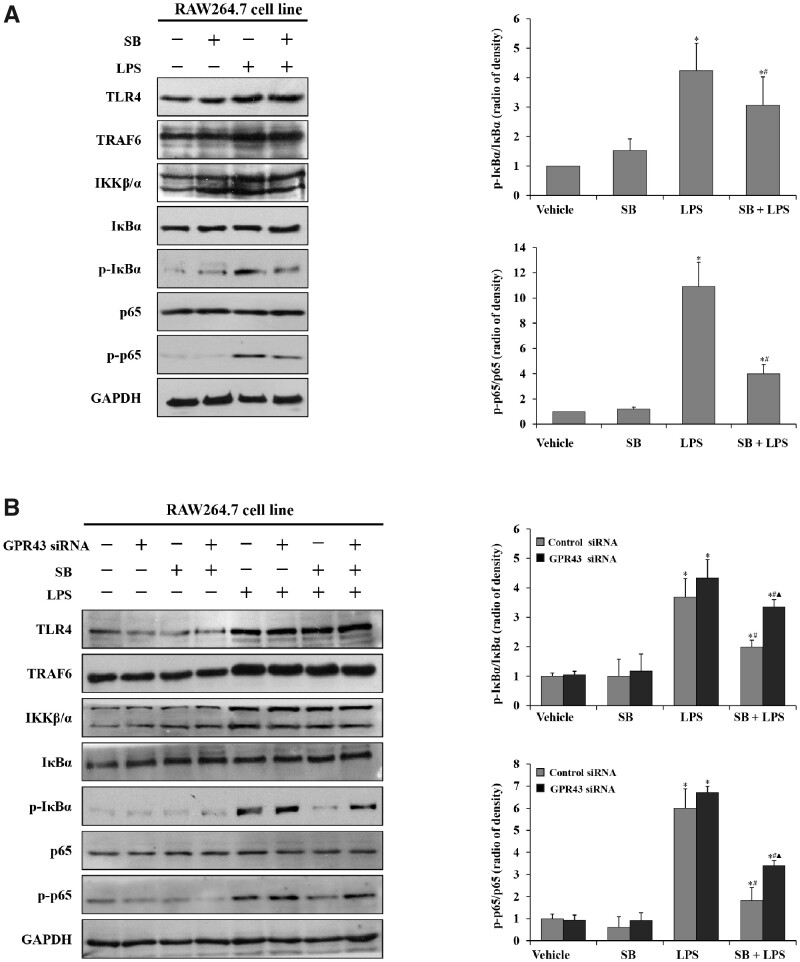
Sodium butyrate/GPR43 signaling repressed macrophage activity via regulating TLR4/NF-κB p65 networks. (A) Expression of TLR4, TRAF6, IKKβ/α, phospho-IκBα, IκBα, phospho-p65, and p65 in RAW264.7 cells was detected by Western blot. Levels of GAPDH are shown as a loading control; relative quantitative evaluation of the Western-blot analysis for phospho-IκBα/IκBα, phospho-p65/p65 expression was obtained using Image-Pro Plus6.0 software. ^*^P <* *0.05 vs vehicle group; ^#^*P *<* *0.05 vs LPS group. Values are expressed as mean* *±* *SD (*n *=* *6 per group). (B) Expression of TLR4, TRAF6, IKKβ/α, phospho-IκBα, IκBα, phospho-p65, and p65 in RAW264.7 cells that were transfected with GPR43 siRNA RNA oligo (GPR43 siRNA) or control siRNA RNA oligo (control siRNA) was detected by Western blot. Levels of GAPDH are shown as a loading control; relative quantitative evaluation of the Western-blot analysis for (phospho-IκBα/GAPDH)/(IκBα/GAPDH), (phospho-p65/GAPDH)/(p65/GAPDH) expression was obtained using Image-Pro Plus6.0 software. ^*^P <* *0.05 vs vehicle control group in the same type of cell; ^#^*P *<* *0.05 vs LPS group in the same type of cell; ▲ *P *<* *0.05 vs SB + LPS group in the control siRNA group. Values are expressed as mean* *±* *SD (*n *=* *6 per group). SB, sodium butyrate.

Given that the TLR4/NF-κB signaling pathway has been associated with LPS-induced liver injury in our previous studies, we prospect that sodium butyrate may alleviate LPS-induced macrophage inflammation through negative regulation of the TLR4/NF-κB signaling pathway. To identify the mechanism, we detected the expression of some key elements in the TLR4/NF-κB signaling pathway. The results showed that TLR4, TRAF6, IKKβ/α, phospho-IκBα, IκBα, phospho-p65, and p65 significantly increased in RAW264.7 cells after treatment with LPS for 6 h (Figure 4A and B), which suggested that LPS-induced liver injury was related to activation of the TLR4/NF-κB signaling pathway. It was also observed that phospho-IκBα and phospho-p65 were not significantly increased in the cells that were treated with sodium butyrate + LPS (Figure 4A), indicating that sodium butyrate might be involved in the negative regulation of the TLR4/NF-κB signaling pathway through interacting with IκBα and p65, resulting in reduced pro-inflammatory-factor production in RAW264.7 cells. In cells that were transfected with GPR43/control siRNA, phospho-IκBα and phospho-p65 were decreased in both sodium butyrate + LPS groups and the control siRNA group was lower than the GPR43 siRNA group, indicating that GPR43 might be involved in sodium-butyrate-mediated regulation of the TLR4/NF-κB p65 signaling pathway (Figure 4B).

### Sodium butyrate increased the binding capacity of GPR43 with β-arrestin-2, as well as β-arrestin-2 with IкBα, in the RAW264.7 cell line

**Figure 5.**
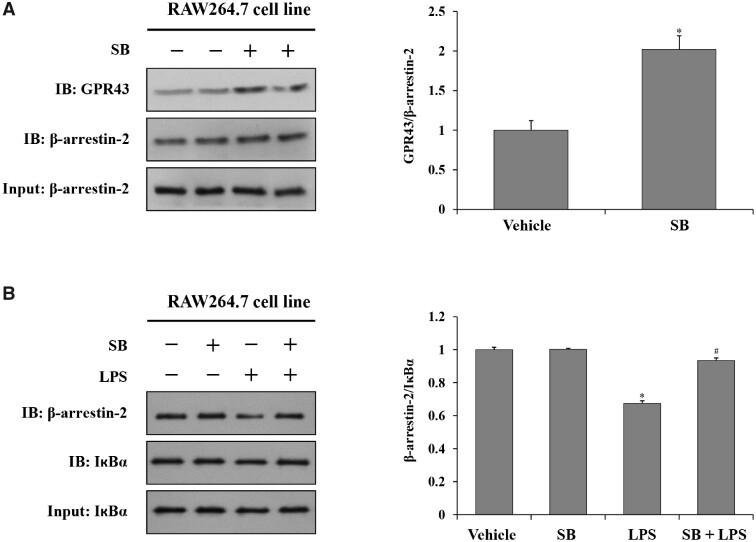
Sodium butyrate increases the binding capacity of GPR43 and β-arrestin-2, β-arrestin-2 and IкBα. (A) Co-IP was used to detect changes associated with pretreatment with sodium butyrate for 30 min of GPR43 and β-arrestin-2 in RAW264.7cells. (B) Co-IP was used to detect changes associated with pretreatment with sodium butyrate for 30 min of β-arrestin-2 and IкBα in RAW264.7cells, followed by lipopolysaccharide treatment for 6 h. Relative quantitative evaluation of the Western-blot analysis for GPR43/β-arrestin-2, β-arrestin-2/IкBα expression was obtained using Image-Pro Plus 6.0 software.^*^P <* *0.05 vs vehicle group; ^#^*P *<* *0.05 vs LPS group; values are expressed as mean* *±* *SD (*n *=* *6 per group, Bonferroni’s comparison post-hoc test). Co-IP, co-immunoprecipitation.

The present study showed that sodium butyrate exerts beneficial effects on LPS-induced liver injury. It was also found that the effect is mediated by GPR43, which later impacts on the TLR4/NF-κB signaling pathway. To obtain a better understanding of the molecular mechanism, we examined whether β-arrestin-2 inhibited in the NF-κB signaling pathway. Co-IP experiments on cell models were used to further investigate the interaction among GPR43, β-arrestin-2, and IкBα. Co-IP results showed that sodium butyrate increased the binding capacity of GPR43 with β-arrestin-2 (Figure 5A), as well as the binding capacity of β-arrestin-2 with IкBα (Figure 5B).

## Discussion

SCFAs are end products of the saccharolytic fermentation of dietary fibers by the anaerobic intestinal microbiota and have a carbon number of between 2 and 6 [32, 33]. The increasing value of SCFA modulation on inflammatory damage and the metabolism of both enteral and parenteral environments has now been recognized. Many studies have indicated that SCFAs exhibit a wide range of functions from immune regulation (IBD [34], autoimmune hepatitis, multiple sclerosis [35], allergy [36], dermatitis [37], etc.) to metabolism (non-alcoholic fatty liver disease [38], obesity, insulin resistance [39, 40], dysregulated bile-acid synthesis [41], hypertensive cardiovascular damage [42], pathological bone loss [43], etc.) in a variety of tissues and organs. Soluble dietary fibers act as an energy source for a selected group of gut bacteria promoting the growth of beneficial microorganisms in the intestine; thus, these fibers are regarded as ‘prebiotics’ in the intestinal microenvironment [44, 45]. In addition to the direct physical action of SCFAs, this ‘microenvironment selection’ may allow further understanding of their role in the suppression of systemic inflammation, pathologic metabolization, and carcinogenesis [46].

The liver harbors the largest proportion of macrophages in all solid organs of the body in response to various toxin exposures. Kupffer cells are resident macrophages that are seeded along sinusoidal endothelial cells and are important scavengers and gatekeepers for the liver microenvironment [47]. In principle, Kupffer cells are sensors of initial tissue damage, given that numerous toxins enter the portal vein every day. Both endogenous ligands generated from injured cells (viral infection, non-alcoholic steatohepatitis, alcoholic liver disease, autoimmune diseases, etc.) and exogenous ligands generated from the gut microbiota (foods, drugs, chemicals, etc.) are under close surveillance once they enter liver sinusoids. By recognizing them, Kupffer cells can become activated and initiate pro-inflammatory cascades. Other liver immune cells [48] include monocyte-derived macrophages, dendritic cells, and ‘innate-like’ T lymphocytes, which are mainly Th17 cells [49, 50]. The majority of liver blood flow is derived from the portal circulation, which is rich in LPS, allowing close contact of microbial products with Kupffer cells. LPS, as the most famous pathogen-associated molecular pattern, activates macrophages by binding TLR4 and cluster of differentiation 14 (CD14), leading to the upregulation of various inflammatory cytokines, including TNF-α, IL-1β, IL-6, IL-12, and IL-18 [51], thus further activating hepatic stellate cells to recruit Kupffer cells and circulating macrophages [52]. Furthermore, recent literature indicates a greater role for Kupffer cells and TNF-α as the main inflammatory mediator that activates the apoptotic pathway [53].

In this study, we established an LPS-induced liver-injury model in female C57BL/6 mice and an LPS-induced liver-inflammation model in the RAW264.7 cell line (macrophages) to investigate the effect of sodium butyrate in attenuating inflammation *in vivo* and *in vitro*. The results confirmed that sodium butyrate reduced the levels of ALT and AST in serum, minimized structural damage, decreased inflammatory infiltration, and reduced liver expression of inflammatory factors induced by LPS. The inflammatory reaction took place in liver macrophages.

Previous studies have shown that butyrate activation of GPR43, the most notable SCFA target, may increase the influx of calcium ions [54, 55] or bind to β-arrestin-2 to modulate the immune response, alleviating the immune response. We established an LPS-induced liver-injury model in female GPR43 KO mice and WT littermates, and LPS-induced inflammation in RAW264.7 cells that were transfected with GPR43 siRNA or control siRNA to investigate the effect of GPR43. Our findings confirmed that deletion of GPR43 partially weakened the effect of sodium butyrate on LPS-induced liver injury in mice and LPS-stimulated inflammation in RAW264.7 cells. We also found that GPR43 is highly expressed in pro-inflammatory macrophages. This proved that GPR43 acts as a butyrate receptor and mediates anti-inflammatory effects.

Several other receptors for SCFAs [56] have also been identified in previous studies, such as G-protein-coupled receptor 41/free fatty acid receptor 3 (GPR41/FFAR3; associated with adiposity and energy homeostasis) [57], GPR109A (associated with immune-cell chemotaxis and activation) [58], and olfactory receptor 78 (associated with the regulation of hormone secretion and blood pressure) [59]. Various studies have shown that these receptors have a similar role to that of GPR43. For example, it was previously found that dietary fiber protects against colitis via GPR43 and GPR109A in a dextran sulfate sodium (DSS)-induced colitis model [16, 60]. In addition, SCFAs also act as histone deacetylase (HDAC) inhibitors and extensively influence inflammatory networks in this way. In another study, HDAC was found to be the underlying mechanism of SCFA-mediated inhibition of LPS-induced TNF-α production in neutrophils [61]. The mechanism could be quite complex, going far beyond what we explored in our study, but the ultimate role of SCFAs points to a disease-alleviating effect.

In the present study, sodium butyrate reduced the expression of inflammatory cytokines induced by LPS and downregulated the TLR4/NF-κB pathway in RAW264.7 cells. NF-κB is a transcription factor that is pivotal in inflammatory responses through regulating gene expression [13, 62]. NF-κB activity is regulated by signal-induced IκB degradation, acetylation and deacetylation of proteins in the NF-κB pathway, as well as NF-κB target-gene manipulation [63]. The interruption of NF-κB by butyrate has been extensively studied in the past. For instance, HDAC inhibition by butyrate alleviates p65 binding to IκBα, which in turn results in the export and reduced expression of NF-κB [64]. Butyrate also rescues the redox machinery, controls reactive oxygen species (ROS) [65], activates PPAR-γ [66], and inhibits IFN-γ signaling [67], resulting in suppression of the NF-κB pathway.

β-Arrestin binds to uncoupled GPCRs at the G-protein as a negative regulatory molecule of GPCR signaling pathways [68]. Upon ligand binding, GPCRs undergo conformational changes that allow them to be recognized by the family of G-protein-coupled receptor kinases and further enhance β-arrestin recruitment and binding to the receptors, which sterically hinders further signaling to G-proteins, leading to the classical phenomenon of receptor desensitization [69]. β-Arrestins also scaffold various proteins to potentiate distinct downstream signaling events through the generation of ‘signalosomes’, including the Hedgehog, Wnt, Notch, and transforming growth factor-beta (TGF-β) pathways, and downstream kinases such as mitogen-activated protein kinase (MAPK) and phosphatidylinositol-3 kinase [70, 71]. Many previous studies have also confirmed that β-arrestin-2 inhibits the NF-κB signaling pathway [22, 27, 72] in several ways. For instance, β-arrestin-2 binds to TAB1, inhibiting the binding of TAB1 to TAK1 and blocking TAK1 phosphorylation and subsequent IκB kinase (IKK) phosphorylation, which ultimately reduces the nuclear transfer of NF-κB [73]. In addition, β-arrestin-2 directly binds with IκB, which inhibits IκB phosphorylation and degradation and reduces NF-κB nuclear transfer and activation [23]. It has also been reported that β-arrestin-2 directly interacts with IKKs, NF-κB-inducing kinase (NIK), and TRAF6 to regulate the inflammatory process [24, 74]. In this study, we demonstrated that, after activation, GPR43 couples with β-arrestin-2, increasing the binding capacity of β-arrestin-2 and IкBα, as measured in co-immunoprecipitation experiments. This binding inhibited the phosphorylation and degradation of IкB, thus inhibiting the free activation and nuclear translocation of NF-κB and partially down-regulating the TLR4/NF-κB signaling pathway to inhibit the inflammatory response.

However, there are several questions that remain to be addressed. First, some researchers believe that butyrate is a double-edged sword for health [75]. ROS and nitric oxide (NO) are produced by butyrate in certain circumstances [76], indicating both suppressive and promoting roles in neutrophil chemotaxis. The effect of butyrate on obesity is also regarded as controversial regarding metabolic aspects. Additionally, there is sufficient evidence to indicate the involvement of other signaling pathways in these SCFA receptors, such as engagement of β-arrestins by GPR109A, gustducin by GPR41, and GPR43 Gq/11-dependent activity inducing the influx of [Ca^++^] into the cytoplasm [77, 78]. The mechanism of the beneficial effect of butyrate on inflammation is more complicated than one possible pathway presented here.

In summary, butyrate, a SCFA, mediates potent anti-inflammatory effects to inhibit the TLR4/NF-κB inflammatory signaling pathway by signaling through GPR43 and β-arrestin-2. Sodium butyrate prevents LPS-induced liver injury through the GPR43/β-arrestin-2/NF-κB signaling pathway. Chemical inhibitors such as sodium butyrate could be promising strategies to prevent and treat LPS-induced liver injury and inflammation. Translating the findings into clinical practice may open up new avenues to pursue interventions in the future.

## Supplementary data

Supplementary data is available at *Gastroenterology Report* online.

## Authors’ contributions

Q.J.L., Y.W.G., and X.Q.W. conceived of and designed the project. Q.J.L., S.M.X., and S.W.T. performed the experiments. H.L.L. and J.J. supplied essential reagents. W.X.Y., K.K.A., and L.L. analysed and interpreted the data. Q.J.L., S.M.X., Y.W.G., and X.Q.W. drafted the manuscript. All authors read and approved the final manuscript.

## Funding

This work was supported by the Science and Technology Foundation of Guangzhou China [201903010099, 201803010018] and the National Natural Science Foundation of China [81470848]. 

## Conflicts of interest

None declared. 

## Supplementary Material

goaa085_Supplementary_Data
