# Supplementary material for: Sodium butyrate protects against lipopolysaccharide-induced liver injury partially via the GPR43/ β-arrestin-2/NF-κB network
Source: Gastroenterol Rep (Oxf). 2020 Nov 22;9(2):154–65. doi: 10.1093/gastro/goaa085 (PMC8128024; doi:10.1093/gastro/goaa085)
Supplement: goaa085_Supplementary_Data [file goaa085_supplementary_data.zip › GR-2020-133R3-Supplementary_Table_1.docx]

Supplementary Table 1. siRNAs’ primer sequences

| **Gene** | **Species** | **Primer sequences** |
| --- | --- | --- |
| GPR43(Ffar2-Mus-798) | mouse | 5’-GCUUCUACAGCAGCAUCUATT  UAGAUGCUGCUGUAGAAGCTT-3’ |
| Negative control | mouse | 5’-UUCUCCGAACGUGUCACGUTT-3’  5’-ACGUGACACGUUCGGAGAATT-3’ |
| GAPDH-420 | mouse | 5’-CACUCAAGAUUGUCAGCAATT  UUGCUGACAAUCUUGAGUGAG-3’ |
