# Supplementary material for: Sodium butyrate protects against lipopolysaccharide-induced liver injury partially via the GPR43/ β-arrestin-2/NF-κB network
Source: Gastroenterol Rep (Oxf). 2020 Nov 22;9(2):154–65. doi: 10.1093/gastro/goaa085 (PMC8128024; doi:10.1093/gastro/goaa085)
Supplement: goaa085_Supplementary_Data [file goaa085_supplementary_data.zip › Supplementary Table 2.docx]

Supplementary Table 2. Primer sequences of above genes

| **Gene** | **Species** | **primer** | **Sequences** |
| --- | --- | --- | --- |
| TNF-α | mouse | Forward | 5’-TTCTGTCTACTGAACTTCGGGGTGATCGGTCC-3’ |
|  |  | Reverse | 5’-GTATGAGATAGCAAATCGGCTGACGGTGTGGG-3’ |
| IL-6 | mouse | Forward | 5’-AGGATACCACTCCCAACAGACCT-3’ |
|  |  | Reverse | 5’-CAAGTGCATCATCGTTGTTCATAC-3’ |
| IL-1β | mouse | Forward | 5’-ATGGCAACTGTTCCTGAACTCAACT-3’ |
|  |  | Reverse | 5’-CAGGACAGGTATAGATTCTTTCCTTT-3’ |
| IL-8 | mouse | Forward | 5’-CATTGCCGGTGGAAATTCCTT-3’ |
|  |  | Reverse | 5’-TCGAGACCATTTACTGCAACAG-3’ |
| COX-2 | mouse | Forward | 5’-TGAGCAACTATTCCAAACCAGC-3’ |
|  |  | Reverse | 5’-GCACGTAGTCTTCGATCACTATC-3’ |
| IFN-γ | mouse | Forward | 5’-ATGAACGCTACACACTGCATC-3’ |
|  |  | Reverse | 5’-CCATCCTTTTGCCAGTTCCTC-3’ |
| β-actin | mouse | Forward | 5’-GGCTGTATTCCCCTCCATCG-3’ |
|  |  | Reverse | 5’-CCAGTTGGTAACAATGCCATGT-3’ |
